# Urban–Rural Differences and Sex‐Specific Cognitive Effects on Autism Symptom Trajectories: A Longitudinal Study of Autistic Children in Taiwan

**DOI:** 10.1002/aur.70193

**Published:** 2026-02-03

**Authors:** Yun‐Ju Chen, Hsiang‐Yuan Lin, Ching‐Lin Chu, Chin‐Chin Wu, Tzu‐Ling Lin, Hsing‐Chang Ni, Jiun‐Horng Liu, Yuh‐Ming Hou, Chung‐Hsin Chiang

**Affiliations:** ^1^ Department of Occupational Therapy and Graduate Institute of Behavioral Sciences Chang Gung University Taoyuan Taiwan; ^2^ Department of Psychiatry Chang Gung Memorial Hospital at Linkou Taoyuan Taiwan; ^3^ Azrieli Adult Neurodevelopmental Centre, Campbell Family Mental Health Research Institute, Centre for Addiction and Mental Health Toronto Ontario Canada; ^4^ Department of Psychiatry, Temerty Faculty of Medicine University of Toronto Toronto Ontario Canada; ^5^ Department of Educational Psychology and Counseling National Pingtung University Pingtung Taiwan; ^6^ Department of Psychology Kaohsiung Medical University Kaohsiung Taiwan; ^7^ Institute of Behavioral Medicine, College of Medicine National Cheng Kung University Tainan Taiwan; ^8^ Department of Psychiatry Chi Mei Hospital at Liouying Tainan Taiwan; ^9^ Department of Psychiatry Ditmanson Medical Foundation Chia‐Yi Christian Hospital Chiayi Taiwan; ^10^ Department of Psychology and Research Center for Mind, Brain and Learning National Chengchi University Taipei Taiwan

**Keywords:** autism, IQ, longitudinal, multilevel modeling, severity change, sex differences

## Abstract

Few longitudinal studies have mapped autism symptom trajectories outside Western contexts. This study aimed to characterize trajectories of autism symptoms, assessed using the Autism Diagnostic Observation Schedule (ADOS), and associated child and family factors among autistic children from two regions in Taiwan that differ by urbanicity. Another aim was to examine the time‐varying effects of children's cognitive abilities on autism symptoms, which remain understudied due to prior reliance on baseline proxies. Children with a confirmed autism diagnosis (*n* = 180, 87.8% male) were followed across three waves of data collection from ages 2 to 11 years. Linear multilevel growth models with random intercepts and slopes were used to estimate symptom trajectories at the total and domain levels of the ADOS. On average, total and social‐affect symptoms increased significantly with age, while restricted and repetitive behaviors (RRBs) remained stable. Children from urban areas showed higher baseline RRBs and smaller increases in social‐affect symptoms compared to those from rural areas. Additionally, children diagnosed under DSM‐5 criteria showed lower baseline symptoms but greater increases in total/social‐affect symptoms over time than their DSM‐IV counterparts. A sex interaction effect was observed in the time‐varying associations between IQ (particularly verbal IQ) and total/social‐affect symptoms, with girls showing stronger negative IQ‐symptom associations. These findings highlight the developmental complexity underlying the manifestation of autism symptoms, particularly at the intersection of sex and cognition. The distinct patterns by urbanicity also underscore the need to mitigate urban–rural disparities in service access to better support autistic children's long‐term outcomes.

## Lay Summary

This study followed 180 autistic children in Taiwan over 9 years and found that autism symptoms changed with age in ways that varied by children's place of residence, sex, and IQ. Children from rural areas showed greater increases in social difficulties, whereas those from urban areas showed higher levels of restricted and repetitive behaviors. Higher IQ was associated with fewer social challenges over time—but only among girls, not boys. These findings highlight the need to consider both individual and environmental factors, such as the child's cognitive abilities, sex, and urban–rural contexts, when assessing and supporting the long‐term development of autistic children.

## 1 Introduction

Differences in social communication and the presence of restricted, repetitive behaviors (RRBs) are hallmark features of autism that can significantly affect daily functioning and well‐being (Oakley et al. 2021). The presentation and impact of core symptoms are highly heterogeneous and evolve across the lifespan, as shown in numerous longitudinal cohort studies (Dellapiazza et al. 2024; Fountain et al. 2012; Lord et al. 2015; Szatmari et al. 2015; Waizbard‐Bartov et al. 2022). Although such studies vary in methodology—including the measures used to assess autism symptoms, follow‐up intervals, study durations, and sampling strategies (Gentles et al. 2024)—converging patterns of stability and change have emerged. In early childhood, the manifestation of core symptoms leading to an autism diagnosis often peaks around ages three to four, after which symptom severity typically decreases or remains stable during the preschool years (Kim et al. 2018; Waizbard‐Bartov and Miller 2023). Upon entering middle childhood, however, rates of change tend to diverge, with symptoms either plateauing or increasing, suggesting a potential turning point in trajectories (Georgiades et al. 2022). However, substantial individual variability in trajectories has been reported and is often associated with child and family characteristics, including the child's cognitive or developmental abilities and parental education (Fountain et al. 2012; Waizbard‐Bartov and Miller 2023). Developmental variability has also been observed across domains; for instance, RRBs tend to increase during the preschool years and plateau in middle childhood (Dellapiazza et al. 2024; Masjedi et al. 2025). Understanding potential developmental pathways across core symptom domains, along with their associated risk and protective factors, has important implications for supporting autistic individuals and their families, as well as for guiding clinicians in providing developmentally and individually tailored services (Kata et al. 2024; Lord et al. 2015).

Despite the growing body of evidence in this area, it is predominantly derived from research conducted in North American and European countries (Gentles et al. 2024), limiting its generalizability to other geographic and cultural contexts. This is particularly relevant for investigations into the contextual factors shaping developmental trajectories, as such factors are inherently influenced by local social, cultural, and service environments and contribute to variation in how autism‐related difficulties are defined, recognized, interpreted, and reported (de Leeuw et al. 2020). Taiwan presents a unique context to explore these factors, where the interplay between a national healthcare system and pronounced regional urbanicity creates distinct service environments. Specifically, the National Health Insurance system has influenced how autism and its related symptoms are recognized and reported, serving as an avenue for early identification and access to services (Huang 2007; Li et al. 2019). However, this national framework is not applied evenly across regions, as a stark urban–rural disparity has consistently been identified as a key factor influencing autism identification, diagnosis, and service utilization (Chen et al. 2008; Chu et al. 2017; Hsu et al. 2023; Li et al. 2019). While this disparity may be attributed to better service access and socioeconomic factors that influence health‐seeking behaviors and awareness of developmental concerns in urban areas (Chen et al. 2008; Lauritsen et al. 2014), the potential influence of urbanicity on the long‐term trajectories of autism symptoms, beyond initial diagnosis, remains largely unexplored. Therefore, studies involving participants recruited from settings that reflect varying levels of urbanicity are warranted to clarify its association with autism symptom development, which could further inform service planning, resource allocation, and policy decisions tailored to diverse community contexts.

Beyond environmental context, the dynamic interplay between cognitive abilities and autism symptoms represents a critical but understudied aspect of developmental trajectories. Rather than serving as a static predictor, cognitive abilities actively shape how children navigate social demands, develop compensatory strategies, and express autism‐related behaviors across development (Mahony et al. 2023; Wolff et al. 2022). Despite this, most trajectory research treats IQ as a fixed baseline characteristic (e.g., Richler et al. 2010; Szatmari et al. 2015; Waizbard‐Bartov et al. 2022), partly due to the challenge of reconciling different cognitive measures used across ages (Simonoff et al. 2020; Solomon et al. 2023) and of addressing floor or ceiling effects in certain cognitive assessments (Chen et al. 2022). Furthermore, a focus on full‐scale IQ alone often obscures the distinct contributions of verbal and non‐verbal abilities (Joseph and Fein 2011). This differentiation is particularly critical, as verbal IQ may function as a protective factor against social communication difficulties, an effect observed more strongly in girls (Harrop, Shire, et al. 2015; Skuse et al. 2009). Taken together, these limitations highlight the need for a more nuanced longitudinal approach that captures both the time‐varying nature of IQ and the distinct roles of its subdomains in shaping the heterogeneity of autism symptom trajectories.

To address these empirical gaps in the study of autism symptom trajectories, the present longitudinal study followed 180 autistic children, recruited from two demographically representative regions of Taiwan—an urban region (Taipei) and a rural region (Chiayi)—across three timepoints over an average span of 6 years. Taipei, a highly resourced metropolis with multiple Early Intervention Reporting and Referral Centers (EIRRCs), contrasts with Chiayi, a less‐densely populated county served by only one such center. This urban**–**rural gradient offers a natural context for examining contextual effects on the developmental course of autism symptoms. Using the Autism Diagnostic Observation Schedule (ADOS; Lord et al. 1999) as a measure of autism symptoms, this study aimed to model growth trajectories of total and domain‐level symptoms across three visits spanning ages 2–11 years. We also examined whether time‐invariant characteristics (i.e., child's sex, residential area, diagnostic criteria, and family socioeconomic status [SES]) and time‐varying characteristics (i.e., full‐scale, verbal, and non‐verbal IQ) are associated with these trajectories.

Based on patterns identified in a prior review (Waizbard‐Bartov and Miller 2023), we hypothesized that, on average, social‐affect symptom trajectories would decline over time, whereas RRB trajectories would remain relatively stable, yet with substantial individual variability in slopes. In addition, drawing on evidence that urbanicity is associated with higher rates of autism identification (Hsu et al. 2023; Lauritsen et al. 2014) and that higher family SES is linked to reductions in core symptoms over time (Fountain et al. 2012; Georgiades et al. 2022; Waizbard‐Bartov et al. 2022), we hypothesized that children from urban areas and/or with more highly educated parents would show more favorable trajectories, likely due to greater awareness of the condition and better access to resources and services. These effects may differ across the ADOS domains, although no specific hypotheses were proposed regarding domain‐level differences. Finally, we hypothesized significant negative associations between IQ and symptoms across both ADOS domains, with these associations expected to be more pronounced in girls, particularly for verbal IQ, based on prior evidence (Harrop, Shire, et al. 2015; Johnson et al. 2021; Skuse et al. 2009).

## 2 Methods

### 2.1 Participants and Procedures

A total of 206 preschool‐aged children (175 males) were recruited through multiple longitudinal research projects that followed the developmental outcomes of autistic children over time. All children were referred from hospital settings for developmental or autism‐related concerns in either Taipei or Chiayi to participate in the study projects, all of which received ethical approval from the review boards at the respective sites. Participants were followed prospectively with a range of behavioral assessments (see *Measures*) conducted across three visits or waves of data collection: at ages 21–58 months in Wave 1 (*M* = 35.1, SD = 7.9), 37–84 months in Wave 2 (*M* = 52.5, SD = 9.8), and 82–142 months in Wave 3 (*M* = 106.2, SD = 12.3). Retention rates were 83.5% from Wave 1 to Wave 2 (172 of 206 retained) and 80.2% from Wave 2 to Wave 3 (138 of 172 retained). All participants with at least one data point were included in the analysis, with 176 (85.4%) providing data at two or more timepoints and 130 (63.1%) completing all three waves.

At each visit, a clinical diagnosis of autism was confirmed by consensus between clinical psychologists and child psychiatrists based on either DSM‐IV criteria (Autistic Disorder, Asperger's Disorder, or Pervasive Developmental Disorder–Not Otherwise Specified) or DSM‐5 criteria for Autism Spectrum Disorder, which was further confirmed by standardized assessment using the Autism Diagnostic Observation Schedule (ADOS). The Autism Diagnostic Interview‐Revised (ADI‐R; Rutter et al. 2003) was included as an additional diagnostic reference starting with the second visit. Of the 206 children recruited, 26 were excluded from analyses as they consistently received only developmental delay diagnoses across all visits. Given our research focus on core autism symptom trajectories, only children with a confirmed autism diagnosis were included in the analyses. This exclusion may limit generalizability to the broader autism phenotype, particularly for children with significant intellectual disabilities who may not meet formal autism criteria despite showing autism‐related features. However, this decision allows for clearer interpretation of trajectories within a diagnostically homogeneous group and aligns with standard practices in autism trajectory research focusing on core symptom change rather than diagnostic stability. This resulted in a final sample of 180 children (158 males), including three children whose diagnoses shifted between autism and developmental delay over time. Baseline characteristics of the final sample by residential area are presented in Table 1.

**TABLE 1:** Baseline characteristics of participants (*N* = 180) by residential area.

	Full sample (*N* = 180)	Taipei (*N* = 141)	Chiayi (*N* = 39)	Chiayi vs. Taipei
	*N* (%)			Odds ratio (*p‐*value)
Male	158 (87.8)	123 (87.2)	35 (89.7)	0.78 (0.79)
Diagnostic criteria				
DSM‐IV	127 (70.6)	88 (62.4)	39 (100)	33.13 (< 0.001***)
DSM‐5	53 (29.4)	53 (37.6)	0 (0)	—
	Mean (SD)			*t*‐Test statistic (*p‐*value)
Chronological age (months)	36.11 (7.81)	37.84 (7.72)	29.95 (4.22)	−8.38 (< 0.001***)
Mental age (months)	24.08 (10.56)	26.06 (11.00)	17.16 (4.15)	−7.74 (< 0.001***)
MSEL‐Developmental Quotient	66.33 (21.44)	68.77 (22.66)	57.69 (13.40)	−3.84 (< 0.001***)
Maternal education (years)	15.07 (2.55)	15.69 (2.06)	12.83 (2.89)	−6.62 (< 0.001***)
Paternal education (years)	15.78 (2.89)	16.29 (2.83)	13.94 (2.34)	−4.49 (< 0.001***)
Family SES (latent factor scores)	0.06 (0.77)	0.22 (0.74)	−0.49 (0.60)	−5.29 (< 0.001***)
ADOS CSS				
Total	6.63 (1.99)	6.67 (1.86)	6.49 (2.43)	−0.43 (0.67)
SA	6.78 (1.93)	6.68 (1.77)	7.15 (2.39)	1.16 (0.25)
RRB	6.65 (2.10)	6.99 (1.73)	5.44 (2.79)	−3.31 (0.002**)

*Note*: **p < 0.01, ***p < 0.001.

It is important to note that our sample included children diagnosed under both DSM‐IV (*n* = 127; 70.6%) and DSM‐5 criteria (*n* = 53; 29.4%). This split, and its confounding with residential region, is a direct result of the study's rolling recruitment timeline (2004–2016), which was structured around the senior author's institutional move from Chiayi to Taipei in 2009. Specifically, participants whose first timepoint of assessment occurred between 2004 and 2009 were recruited from the rural Chiayi region. Participants whose first timepoint of assessment occurred between 2009 and 2016 were recruited from the urban Taipei region. All subsequent follow‐up assessments for a given participant were conducted at their original site of recruitment. Because the transition to DSM‐5 occurred in 2013, all participants from the Chiayi site were recruited under DSM‐IV criteria, while the Taipei site includes participants recruited under both DSM‐IV (from 2009 to 2013) and DSM‐5 (from 2013 to 2016). Consequently, the DSM‐5 cohort was recruited exclusively from Taipei, had more years of maternal education, and showed lower baseline developmental quotients (DQ), mental age, and ADOS SA and total scores (see Table S1). We addressed this potential confound by including DSM cohort as a covariate in all analyses and conducting sensitivity analyses restricted to the DSM‐IV cohort (see Section 2.2.4 for details).

### 2.2 Measures

#### 2.2.1 Autism Symptoms

The ADOS (Lord et al. 1999) is a semi‐structured, standardized observational assessment widely used for the diagnostic evaluation of autism. In the current study, Calibrated Severity Scores (CSSs; Gotham et al. 2009) for the overall score, as well as the Social Affect (SA) and Restricted and Repetitive Behaviors (RRB) domains, were used to quantify autism symptoms. CSSs range from 1 to 10, with higher scores indicating greater symptom severity. Module selection was based on participants' expressive language and developmental level, with Modules 1 and 2 used at Waves 1 and 2, and Modules 1–3 at Wave 3.

#### 2.2.2 Cognitive and Developmental Abilities

At Wave 1, children's developmental abilities were assessed using the Mullen Scales of Early Learning (MSEL; Mullen 1995). At Waves 2 and 3, various IQ measures that had been translated into Chinese and validated in Taiwan were administered based on each participant's chronological and mental age, including the Wechsler Preschool and Primary Scale of Intelligence‐Revised (WPPSI‐R; Wechsler 2000), Fourth Edition (WPPSI‐IV; Wechsler 2013), and the Wechsler Intelligence Scale for Children–Fourth (WISC‐IV; Wechsler 2007) and Fifth Editions (WISC‐V; Wechsler 2018). Because cognitive ability (IQ) was treated as a time‐varying variable in the current analysis, harmonization across measurement metrics was needed. For each participant and Wechsler measure, age equivalents derived from subtest raw scores were averaged to compute a global age equivalent, which was then divided by chronological age to yield a ratio IQ, analogous to the MSEL Developmental Quotient (DQ). These ratio IQs served as a common metric to estimate standardized IQ scores for participants without a Wechsler‐based score, using a regression model that included their concurrent MSEL DQ and individual average ratio IQ across available waves as predictors. This procedure was applied to derive Full‐Scale IQ (FSIQ), Verbal IQ (VIQ), and Nonverbal IQ (NVIQ) based on the ratio of mental to chronological age (i.e., ratio IQ) using the relevant subscales. The descriptive statistics of standardized IQ and ratio IQ scores are presented in Table S2.

#### 2.2.3 Family SES

Family demographic data included maternal and paternal years of education, as well as the occupation of the primary household income earner. To align with Taiwan's education system, parental education levels were coded as follows: 1 = less than 12 years (did not complete high school), 2 = 12–15 years (high school or vocational school graduate; some college or junior college), 3 = 16 years (bachelor's degree), and 4 = more than 16 years (graduate education). The occupation of the primary household income earner was coded using the Taiwanese Occupational Prestige Scale developed by Hwang (2008): 1 = manual labor and low‐skilled occupations (e.g., construction workers, street vendors), 2 = skilled trades and personal care work (e.g., mechanics, chefs), 3 = clerical and service‐related jobs (e.g., administrative assistants, retail workers), 4 = technical and semi‐professional positions (e.g., nurses, engineers, teachers), and 5 = high‐status professional and managerial roles (e.g., physicians, professors). Higher scores indicate greater occupational prestige and are generally associated with higher family SES as perceived by the Taiwanese public. Confirmatory factor analysis was conducted to estimate latent family SES scores, with the three indicators specified to load onto a single factor standardized to have a mean of 0 and a variance of 1. The model demonstrated excellent fit (CFI = 0.99, TLI = 1.00, RMSEA < 0.001), with standardized factor loadings of 0.79 for maternal education, 0.90 for paternal education, and 0.76 for occupational prestige of the primary household income earner.

#### 2.2.4 Statistical Analysis

We used multilevel growth modeling to estimate the longitudinal trajectories of autism symptoms (total and domain‐level CSS). Multilevel growth models are well suited for handling data with varying assessment intervals across individuals, incorporating fixed effects (population averages), random effects (individual variability), and time to estimate between‐person variability in within‐person change (Curran et al. 2012). Individual data were clustered within three measurement occasions (Waves 1–3), with time coded as chronological age ranging from 21 to 142 months and centered at 21 months. All models were conducted in R using the lme4 (version 1.1–37) and lmerTest (version 3.1–3) packages. Full information maximum likelihood estimation was applied to obtain robust estimates based on all available data, accounting for varying missingness across waves.

We first estimated a series of unconditional growth models to establish average trajectories of autism symptoms. Model 0, a baseline random intercept‐only model, was used to calculate intraclass correlation coefficients (ICCs) and provide model fit indices for comparison with more complex models. In Model 1, a fixed slope was added to estimate the average linear rate of change. Model 2 further included a random slope to capture individual variability in the rate of change and the covariance between the random intercept and slope. Model fit indices, including the log‐likelihood (LL), Akaike's information criterion (AIC), and Bayesian information criterion (BIC), were evaluated for model selection. Likelihood ratio tests (LRTs) were used to compare nested models, with the more complex model preferred when the added parameters significantly improved model fit.

Next, conditional growth models were fitted by adding time‐invariant covariates to identify factors contributing to variability in autism symptom trajectories. The time‐invariant covariates include sex (1 = male, 0 = female), residential region (1 = Taipei, 0 = Chiayi), and family SES latent factor scores. To examine time‐varying effects of IQ on autism symptoms, we included a person‐mean centered IQ variable at Level 1 (i.e., each person's IQ at a given wave minus their average IQ across waves) to capture within‐person effects. The grand‐mean centered person‐level mean IQ was included at Level 2 to model between‐person effects. FSIQ, VIQ, and NVIQ were tested as time‐varying covariates in separate models. Study cohort membership (1 = DSM‐5, 0 = DSM‐IV) was included in all conditional models to account for differences among participants recruited under different DSM criteria. A sensitivity analysis was also conducted on the DSM‐IV cohort only (i.e., excluding the DSM‐5 cohort) to assess the robustness of the conditional effects.

## 3 Results

### 3.1 Unconditional Trajectories of Autism Symptoms

The results of the unconditional growth models for total, SA, and RRB CSSs are presented in Table 2. The ICCs, ranging from 0.23 to 0.27, suggest that 73%–77% of the total variance is attributable to within‐person variability over age. LRTs indicated that Model 1 with fixed slopes was sufficient for SA CSS, while Model 2, which allowed for random slopes, provided a significantly better fit than Model 1 for total and RRB CSS, suggesting substantial variability in slopes. For consistency, Model 2 was selected for total, SA, and RRB CSSs in subsequent analyses. The slope parameters indicate a significant increase with age in total CSS (Est. = 0.11, SE = 0.04, *p* = 0.007) and SA CSS (Est. = 0.12, SE = 0.04, *p* = 0.001), but no significant change for RRB CSS (Est. = −0.07, SE = 0.05, *p* = 0.124). Significant correlations between the random intercept and slope were observed across ADOS total and domain scores (*r* = −0.23 to −0.61), indicating that lower initial levels of autism symptoms were moderately associated with greater increases with age.

**TABLE 2:** Unconditional multilevel growth models of ADOS total and domain scores.

	Model	Intercept		Slope		Intercept–slope correlation	AIC	BIC	LRT χ^2^ (*p*)
		Mean (SE)	Variance	Mean (SE)	Variance				
Total ICC = 0.268	1	6.40 (0.16)	1.22	0.10** (0.04)	—	—	1812.0	1828.2	7.62 (0.014*)
	2	6.41 (0.16)	1.88	0.11** (0.04)	0.07	−0.48	1808.4	1832.7	
SA ICC = 0.255	1	6.52 (0.16)	1.13	0.12*** (0.03)	—	—	1875.8	1892.2	2.02 (0.26)
	2	6.51 (0.16)	1.22	0.12** (0.04)	0.03	−0.23	1877.8	1902.3	
RRB ICC = 0.229	1	6.67 (0.17)	1.13	−0.06 (0.04)	—	—	1867.3	1883.5	22.73 (< 0.001***)
	2	6.70 (0.17)	2.25	−0.07 (0.05)	0.13	−0.61	1848.6	1872.9	

*Note*: *p < 0.05, **p < 0.01, ***p < 0.001.

### 3.2 Baseline Predictors of Autism Symptom Trajectories

As shown in Table 3, results from the conditional growth models revealed that children residing in urban areas (i.e., Taipei) had significantly higher baseline RRB symptoms than those in rural areas (*β* = 2.39, SE = 0.44, *p* < 0.001). Children diagnosed under DSM‐5 criteria had significantly lower ADOS total and domain scores compared to those diagnosed under DSM‐IV criteria (*β* = −1.17 to −1.85, SE = 0.36 to 0.40, all *p* < 0.01). No significant main effects were observed for sex or family SES.

**TABLE 3:** Conditional multilevel growth models of ADOS total and domain scores.

	Total		Social affect		Repetitive and restricted behaviors	
	Main effect (SE)	Slope interaction effect (SE)	Main effect (SE)	Slope interaction effect (SE)	Main effect (SE)	Slope interaction effect (SE)
Time‐invariant covariates						
Male	−0.41 (0.48)	0.15 (0.11)	−0.59 (0.48)	0.15 (0.10)	0.38 (0.52)	−0.05 (0.15)
Urban	1.18 (0.41)	−0.37 (0.09)***	0.28 (0.41)	−0.31 (0.09)***	2.39 (0.44)***	−0.13 (0.12)
SES	0.08 (0.22)	−0.02 (0.05)	0.13 (0.22)	−0.02 (0.05)	−0.09 (0.24)	0.08 (0.07)
DSM‐5	−1.85 (0.36)***	0.38 (0.09)***	−1.75 (0.37)***	0.35 (0.08)***	−1.17 (0.40)**	0.23 (0.12)

	Main effect (SE)	Main effect (SE)	Main effect (SE)
Time‐varying covariates^a^			
FSIQ			
Person‐level average (Level 2)	−0.03 (0.00)***	−0.02 (0.00)***	−0.03 (0.00)***
Person‐mean‐centered (Level 1)	−0.04 (0.03)	−0.05 (0.03)	0.03 (0.03)
FSIQ*male	0.05 (0.03)*	0.06 (0.02)*	−0.01 (0.03)
FSIQ*Urban	−0.02 (0.02)	−0.01 (0.02)	−0.02 (0.02)
FSIQ*SES	−0.00 (0.01)	−0.01 (0.01)	0.03 (0.02)
VIQ			
Person‐level average (Level 2)	−0.03 (0.00)***	−0.02 (0.00)***	−0.03 (0.00)***
Person‐mean‐centered (Level 1)	−0.02 (0.02)	−0.04 (0.02)*	0.02 (0.02)
VIQ*male	0.04 (0.02)*	0.04 (0.02)**	−0.01 (0.02)
VIQ*Urban	−0.02 (0.01)	−0.01 (0.01)	−0.01 (0.02)
VIQ*SES	−0.01 (0.01)	−0.01 (0.01)	0.01 (0.01)
NVIQ			
Person‐level average (Level 2)	−0.03 (0.01)***	−0.02 (0.01)***	−0.03 (0.01)***
Person‐mean‐centered (Level 1)	−0.05 (0.04)	−0.09 (0.04)*	0.06 (0.05)
NVIQ*male	0.06 (0.04)	0.08 (0.04)*	−0.04 (0.04)
NVIQ*Urban	−0.01 (0.02)	0.01 (0.02)	−0.02 (0.03)
NVIQ*SES	−0.01 (0.02)	−0.03 (0.02)	0.04 (0.02)

^a^ FSIQ, VIQ, and NVIQ were included in separate models.

* *p* < 0.05.

** *p* < 0.01.

*** *p* < 0.001.

**FIGURE 1:**
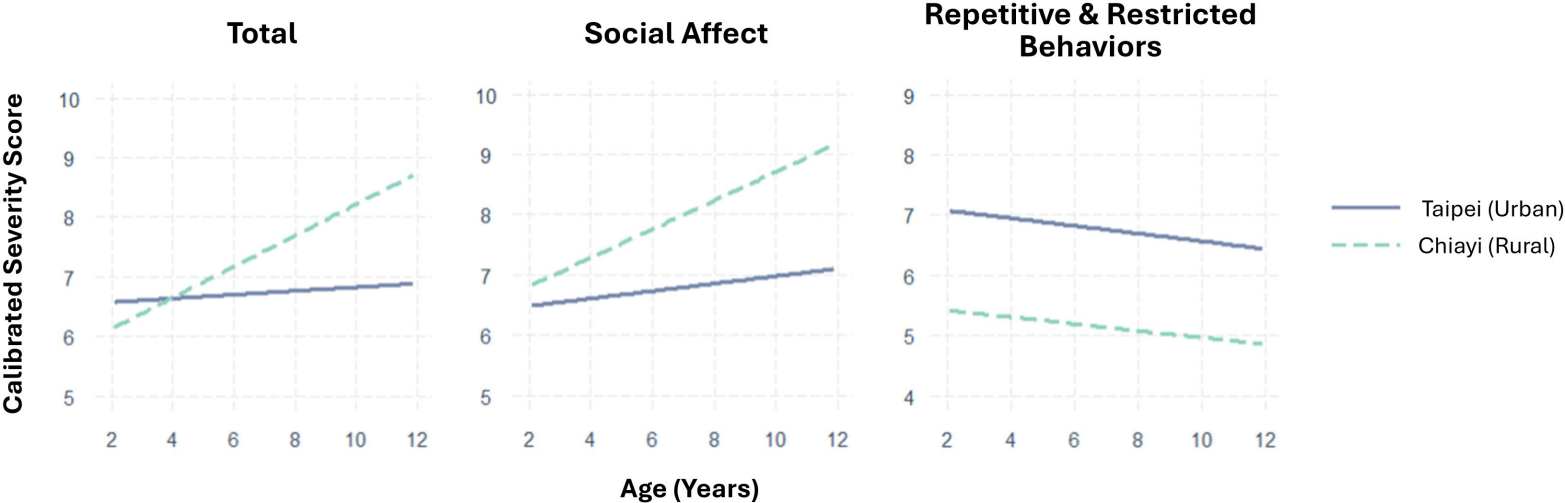
Model‐estimated linear trajectories of ADOS total and domain scores for children in urban (Taipei) and rural (Chiayi) regions (unadjusted by other time‐invariant variables).

Regarding slope interaction effects, children from rural areas showed greater increases in symptoms over age in ADOS total (*β* = −0.37, SE = 0.09, *p* < 0.001) and SA domain scores (*β* = −0.31, SE = 0.09, *p* < 0.001) compared to those from urban areas (see Figure 1). A significant slope interaction effect was also observed for cohorts under different DSM criteria, with children diagnosed under DSM‐5 criteria showing greater increases in symptoms over age in ADOS total (*β* = 0.38, SE = 0.09, *p* < 0.001) and SA domain scores (*β* = 0.35, SE = 0.08, *p* < 0.001). No significant slope interaction effects were found for the RRB domain.

### 3.3 Time‐Varying Associations Between IQ and Autism Symptoms

**FIGURE 2:**
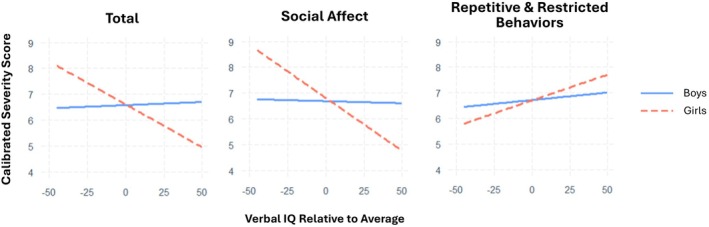
Predicted ADOS total and domain scores as a function of time‐varying verbal IQ, by sex. The *x*‐axis represents a child's Verbal IQ at a given timepoint relative to their own average VIQ across all waves.

Significant main effects were observed for person‐level average FSIQ, VIQ, and NVIQ (Level 2; *β* = −0.02 to −0.03, all SE < 0.005, *p* < 0.001), indicating that children with higher average IQ across time showed lower autism symptom severity at the total and domain levels. In contrast, within‐person fluctuations in IQ (person‐mean‐centered IQ; Level 1) were significantly associated with symptom severity only in the SA domain (*β* = −0.04 for VIQ and −0.09 for NVIQ, SE = 0.02 and 0.04, respectively; both *p* < 0.05), suggesting that increases in IQ relative to a child's own average were associated with concurrent reductions in SA symptoms. Notably, significant cross‐level interactions between sex and IQ were observed for total scores (*β* = 0.05 for FSIQ and 0.04 for VIQ; SE = 0.03 and 0.02, respectively; both *p* < 0.05) and SA domain scores (*β* = 0.04 to 0.08, SE = 0.02 to 0.04, *p* < 0.05 for all IQ types), whereas the interaction was non‐significant for the RRB domain. Given the consistent direction of effects across IQ types and the slightly larger effects observed for VIQ, we plotted Figure 2 to illustrate that the negative associations between VIQ and autism symptoms were more pronounced in girls than in boys. No cross‐level interactions were observed between IQ and ADOS scores by urbanicity or SES.

### 3.4 Sensitivity Analysis: Conditional Models in the DSM‐IV Cohort Only

When the same conditional trajectory models were applied to the DSM‐IV cohort only, the results (presented in Table S3) indicated that all the time‐invariant and time‐varying effects observed in the full sample held, supporting the robustness of these effects. Additional effects were also observed: girls showed greater reductions in total and SA symptom severity with age compared to boys (*β* = 0.32 and 0.29, SE = 0.11 and 0.10, respectively; both *p* < 0.01; see Figure S2). Children from families with higher family SES showed higher SA symptoms at baseline (*β* = 0.68, SE = 0.32, *p* = 0.038). A cross‐level interaction effect of urbanicity was observed for VIQ on total and SA symptoms (*β* = −0.04 and –0.03, both SEs = 0.02, *p* < 0.05), suggesting that the negative association between VIQ and symptom severity was stronger among children from Taipei than those from Chiayi.

## 4 Discussion

This study contributes evidence beyond Western contexts by examining the developmental trajectories of autism symptoms among autistic children across two regions of Taiwan that differ in urbanicity. Our results revealed that while RRBs remained stable, total and social‐affect symptoms tended to increase significantly with age. This finding of domain‐specific change, particularly the stability in RRBs, is consistent with some previous longitudinal research (Dellapiazza et al. 2024; Richler et al. 2010). The increasing trend in social‐affect symptoms observed in our study aligns with findings from Waizbard‐Bartov et al. (2022) but contrasts with the improving trend reported by Dellapiazza et al. (2024). These previous studies and the current one followed autistic children over a similar age range into middle childhood but yielded variable findings, highlighting the heterogeneity of developmental trajectories in autism and the importance of examining symptom domains separately.

Beyond these average trends, we identified key factors that explain the substantial individual variability in symptom trajectories. One of the most novel findings relates to urbanicity. Children in the urban Taipei setting showed higher baseline RRBs compared to their peers in rural Chiayi. While the current data are limited in clarifying its underlying mechanisms, prior research on urbanicity and autism suggests that stressors such as pollution and sensory overstimulation may exacerbate symptom expression (Evans et al. 2018; Lauritsen et al. 2014), with our findings indicating that this effect may be particularly pronounced for RRBs. The higher RRB scores observed in urban settings may reflect multiple contributing factors, such as increased caregiver awareness of atypical behaviors (Barber et al. 2022), or greater environmental demands that make RRB more apparent as efforts to conform to behavioral norms or as coping mechanisms in response to unpredictable changes (de Leeuw et al. 2020; Vanolo 2023). However, our results did not indicate any differences in the change of RRBs between children residing in urban versus rural areas, suggesting that while urbanicity is associated with higher baseline severity, it may play a minimal role in the rate of change in RRBs over time.

In contrast, although baseline social‐affect symptoms did not differ by urbanicity, children residing in rural areas showed more pronounced increases in these symptoms across age. While the specific mechanisms underlying this association cannot be confirmed with the current data, the effect of urbanicity remained significant even after controlling for family SES, which was not a significant predictor. This suggests that regional factors beyond socioeconomic disparities, such as differences in access to services, may be driving the observed variation in symptom trajectories. Previous studies have linked urbanicity primarily to autism symptom levels or rates of autism identification and diagnosis (e.g., Chen et al. 2008; Lauritsen et al. 2014). To our knowledge, this is the first study to demonstrate differences in the rate of change in autism symptoms based on urbanicity. Our findings suggest that the greater availability of resources in urban areas such as Taipei, where access to early diagnostic and intervention services is more readily available, may serve as a protective factor against worsening social communication symptoms over time. These results highlight the need for approaches that address urban–rural disparities in service access, including the use of telehealth, to better support children's developmental outcomes (Antezana et al. 2017; Ashburner et al. 2016).

It is worth noting a significant cohort effect on symptom trajectories: children diagnosed under DSM‐5 criteria, who were recruited after 2013, showed lower autism symptoms at baseline but greater increases particularly in social‐affect symptoms compared to those diagnosed under DSM‐IV criteria. Although unexpected, this pattern may be partly attributable to the lower baseline ADOS scores in the DSM‐5 cohort, which may have allowed more room for increases over time. In addition, our children diagnosed under DSM‐5 criteria were exclusively recruited from Taipei. Although urbanicity was generally associated with improving trajectories (i.e., smaller increases) in social‐affect symptoms, the DSM‐5 cohort showed greater increases in symptoms. This suggests that the more favorable trajectories observed among children from the urban region were largely driven by the DSM‐IV cohort. It is possible that the DSM‐IV cohort included children who were more verbally or cognitively able at baseline, as reflected in significantly higher baseline mental ages (see Table S1), and who therefore may have had greater potential to show more favorable trajectories (i.e., symptom stabilization or attenuation) in social‐communication difficulties, as indicated by prior research (Chen et al. 2024; Waizbard‐Bartov and Miller 2023). While the DSM cohort effect was not the primary focus of our investigation, as it reflects a methodological caveat, the observed effect may indicate the need for a new generation of autism cohort studies that move beyond the existing evidence, which is largely based on children diagnosed during the DSM‐IV era (Kata et al. 2024). It is also recommended to adopt dimensional approaches, which are better supported under DSM‐5, to capture individual variability in the quantity and quality of specific social‐communication difficulties and RRBs, thereby allowing for more nuanced sample characterization and behavioral phenotyping (Grzadzinski et al. 2013).

We also note that two time‐invariant covariate effects were identified in the DSM‐IV cohort only: autistic boys showed increasing trajectories in total and social‐affect symptoms over time, and children from families with higher SES had higher levels of social‐affect symptoms at baseline. Since the two cohorts did not differ in sex ratio, the observed sex difference may reflect diagnostic discordance for females across DSM versions, with girls more likely to meet DSM‐IV but not DSM‐5 criteria (Mazurek et al. 2017). As a result, the DSM‐5 cohort may include girls with more pronounced symptoms, contributing to trajectories resembling those of boys. In contrast, the DSM‐IV cohort likely captured a broader range of female presentations, including those with subtler social‐affect symptoms, which may explain the observed sex differences. Nonetheless, these interpretations should be made with caution given the potential confounding of region and DSM cohort in the current study. As for the differences related to family SES, this may largely stem from the fact that the DSM‐5 cohort was recruited exclusively from Taipei, reflecting a more homogeneous SES distribution. This homogeneity may obscure SES‐related effects when the DSM‐5 cohort is included in the full sample. These cohort‐specific effects underscore the importance of considering cohort differences when examining autism symptom trajectories, particularly in studies with rolling enrollment designs, as such differences may reflect clinically meaningful variation.

Regarding the time‐varying effect of IQ on autism symptoms, a significant cross‐level interaction with sex was consistently observed for full‐scale, verbal, and non‐verbal IQ in relation to social‐affect symptoms, but not RRBs. Specifically, higher IQ was significantly associated with lower social‐affect symptoms over time in girls, whereas this association was not observed in boys. This finding is consistent with prior studies of a general population cohort in the United Kingdom, which reported a negative association between verbal IQ and social communication difficulties in girls—but not in boys—with this association remaining stable over time (Robinson et al. 2011; Skuse et al. 2009). Our observations among children referred for autism diagnostic evaluation in early childhood further suggest that while this moderating effect of sex was present across IQ types, it was most robustly and consistently driven by verbal IQ. The observed absence of sex differences in the time‐varying association between IQ and RRBs also aligns with previous cross‐sectional evidence from a clinical sample (Harrop, Gulsrud, et al. 2015).

Our observation of a sex‐differential association between IQ and social‐affect symptoms may be consistent with camouflaging behaviors more commonly reported among autistic females (Dean et al. 2017; McQuaid et al. 2022; Wood‐Downie et al. 2021), whereby higher cognitive abilities could support the use of compensatory strategies in social interactions (Lehnhardt et al. 2016; Livingston et al. 2019). During diagnostic evaluations, social difficulties in children with higher cognitive abilities, particularly among girls, may be less overt and therefore more difficult for clinicians to detect, potentially reflecting sex‐specific phenotypes and/or the use of camouflaging strategies (Dworzynski et al. 2012; Howe et al. 2015; Wood‐Downie et al. 2021). Previous research indicates that autistic girls tend to demonstrate greater reductions in core symptoms than boys (Waizbard et al. 2025), alongside increases in IQ (Waizbard‐Bartov et al. 2024), supporting our observation that the association between core symptoms and IQ may be more pronounced among girls. Further, our examination of different IQ indices indicated a slightly stronger sex interaction for the association between verbal IQ and social‐affect symptoms. Our longitudinal findings are consistent with prior cross‐sectional results reported by Skuse et al. (2009), which showed that verbal IQ was protective for females, but not for males, in relation to social communication traits, while no such association was found for nonverbal or full‐scale IQ. Higher verbal IQ may be associated with better conversational mimicry, narrative skills, and the use of scripted social responses to mask social‐communication differences, which are more commonly observed in females (Hull et al. 2020; Parish‐Morris et al. 2017). All these findings highlight the complex interplay between cognitive differences and the nuanced, sex‐specific presentations of autism across development, underscoring the need for future efforts to develop sex‐sensitive, developmentally‐informed tools and strategies to reduce service access inequities arising from such variability, particularly for females (Lai et al. 2023). Given the differential results between social‐affect difficulties and RRBs, we also emphasize the importance of domain‐level analyses to better characterize the nuanced developmental trajectories of autism symptoms and their associated factors.

## 5 Limitations and Future Directions

The strengths of this study should be weighed against several limitations. First, each participant contributed at most three data points, which limited our ability to test for non‐linear trajectories across age. Second, although retention rates at each wave were relatively high (~85%), and the majority of participants (~63%) completed all three waves, the potential influence of attrition on the estimation of trajectory parameters and conditional effects cannot be ruled out, despite the use of full information maximum likelihood estimation to handle missing data. Further, our participants were drawn from multiple research projects that shared data collection protocols for the measures of interest, but were conducted under different DSM criteria due to the extended time span of the study (2004–2016). As detailed in the Methods, the sequential nature of recruitment across the two sites and the introduction of DSM‐5 in 2013 resulted in the DSM‐5 cohort being exclusively recruited from the urban region (Taipei). This creates an inherent entanglement between residential region, DSM criteria, and the historical time period of recruitment. While efforts have been made to account for these differences through covariate adjustment and sensitivity analyses restricted to the DSM‐IV cohort, we acknowledge that these factors cannot be fully disentangled, limiting the ability to isolate the independent effects of region and DSM cohort. This warrants caution in interpreting the results and underscores the need for future longitudinal research to explicitly account for DSM cohort membership and recruitment timelines when applicable. We also note that the sample size of our female group was modest (*n* = 22; 55 data points in total), which may have limited the statistical power to detect sex‐specific effects and warrants cautious interpretation of any observed group differences. Moreover, the harmonization of IQ measures represents a statistical approximation that minimizes discontinuities in the developmental metric but may not fully capture conceptual equivalence across instruments. Therefore, while the analysis does not seek to model longitudinal changes in IQ per se but rather to treat IQ as a time‐varying covariate, the results should nevertheless be interpreted with caution, as they may partly reflect measure‐related differences rather than developmental change. Finally, using residential areas as a proxy for urbanicity may be limited in capturing regional variations in factors such as access to services, provider availability, and socioeconomic disparities, which can change over time and potentially influence developmental outcomes. Future investigations into developmental trajectories should incorporate contextual factors through finer‐grained measures of differential opportunities and barriers within environments, to better evaluate and promote person‐environment fit that supports positive outcomes for autistic people (Lai et al. 2020; Mailick et al. 2025).

## 6 Conclusion

In modeling the developmental trajectories of autism symptoms among 180 autistic children in Taiwan, the results revealed substantial variability in total and domain‐level autism symptoms. Notably, children residing in rural areas showed greater increases with age in social‐affect symptoms, even after accounting for individual and family factors such as SES. Our examination of time‐varying associations between IQ and autism symptoms further revealed sex as a significant moderator, with stronger negative associations between IQ and social‐affect symptoms observed in girls. Our findings suggest a complex interplay between individual characteristics (e.g., sex, cognition) and environmental contexts (e.g., urbanicity) in shaping the developmental trajectories of autistic symptom manifestation. This highlights the need to move beyond one‐size‐fits‐all approaches and toward policies that address resource inequities between rural and urban regions. Such policies, combined with nuanced clinical strategies that are tailored to optimize the person‐environment fit, are essential for supporting autistic people in developing to their fullest potential.

## Funding

This work was supported by National Science and Technology Council of Taiwan, 111‐2410‐H‐004‐101‐MY3, 102‐2410‐H‐004‐044‐MY3.

## Ethics Statement

Ethics approval for this study was obtained from the Institutional Review Board of Ditmanson Medical Foundation Chia‐Yi Christian Hospital (IRB No. 100018) and the Research Ethics Committee of National Chengchi University (NCCU‐REC No. 201512‐I056).

## Conflicts of Interest

The authors declare no conflicts of interest.

## Supporting information


**Data S1:** aur70193‐sup‐0001‐Supinfo.docx. **Table S1**. Baseline sample characteristics for children in the DSM‐IV and DSM‐5 cohorts
**Table S2:** Descriptive statistics of the standardized IQ and ratio IQ scores
**Table S3:** Conditional multilevel growth models of ADOS total and domain scores for the DSM‐IV cohort only
**Figure S1:** Model‐estimated trajectories of ADOS total and domain scores
**Figure S2:** Model‐estimated linear trajectories of ADOS total and domain scores by sex for the DSM‐IV cohort onl.

## Data Availability

The data that support the findings of this study are available from the corresponding author upon reasonable request.
