# Supplementary material for: Urban–Rural Differences and Sex‐Specific Cognitive Effects on Autism Symptom Trajectories: A Longitudinal Study of Autistic Children in Taiwan
Source: Autism Res. 2026 Feb 3;19(3):e70193. doi: 10.1002/aur.70193 (PMC12996854; doi:10.1002/aur.70193)
Supplement: Supplementary file 1 — Data S1: aur70193‐sup‐0001‐Supinfo.docx. Table S1. Baseline sample characteristics for children in the DSM‐IV and DSM‐5 cohorts Table S2: Descriptive statistics of the standardized IQ and ratio IQ scores Table S3: Conditional multilevel growth models of ADOS total and domain scores for the DSM‐IV cohort only Figure S1: Model‐estimated trajectories of ADOS total and domain scores Figure S2: Model‐estimated linear trajectories of ADOS total and domain scores by sex for the DSM‐IV cohort onl. [file AUR-19-0-s001.docx]

**Supplementary Tables and Figures**

Table S1. Baseline sample characteristics for children in the DSM-IV and DSM-5 cohorts

|  | DSM-IV Cohort  (N=127) | DSM-5 Cohort (N=53) | DSM-5 vs. DSM-IV Cohorts |
| --- | --- | --- | --- |
|  | N (%) | | Odds ratio  (*p-*value) |
| Male | 111 (87.4) | 47 (88.7) | 1.13 (.81) |
| Residential Area |  |  |  |
| *Taipei* | 88 (69.3) | 53 (100) | 47.76 (.007^**^) |
| *Chiayi* | 39 (30.7) | 0 (0) | .02 (.007^**^) |
|  | Mean (SD) | | T-test statistic  (*p-*value) |
| Chronological age (months) | 36.70 (8.63) | 34.71 (5.24) | 1.89 (.061) |
| Mental age (months) | 25.25 (11.37) | 21.38 (7.82) | 2.60 (.010^*^) |
| MSEL-Developmental Quotient | 68.37 (21.91) | 61.56 (19.66) | 2.04 (.044^*^) |
| Maternal education (years) | 14.77 (2.59) | 15.72 (2.35) | -2.21 (.029^*^) |
| Paternal education (years) | 15.48 (2.45) | 16.45 (3.64) | -1.71 (.092) |
| Family SES (latent factor scores) | .00 (.73) | .21 (.83) | -1.66 (.099) |
| ADOS CSS |  |  |  |
| *Total* | 6.99 (2.06) | 5.77 (1.54) | 4.35 (<.001^***^) |
| *SA* | 7.19 (1.99) | 5.81 (1.36) | 5.35 (<.001^***^) |
| *RRB* | 6.69 (2.25) | 6.57 (1.72) | .35 (.724) |

Note. ^*^*p* < .05, ^**^*p* < .01, ^***^*p* <.001.

Table S2. Descriptive statistics of the standardized IQ and ratio IQ scores

|  |  | Full Scale | | | Verbal | | | Nonverbal | | |
| --- | --- | --- | --- | --- | --- | --- | --- | --- | --- | --- |
| Measure |  | **IQ** | **ratio IQ** | ***r*** | **IQ** | **ratio IQ** | ***r*** | **IQ** | **ratio IQ** | ***r*** |
| MSEL | *Mean* | 67.59 | 69.42 | .997 | 62.31 | 61.74 | .999 | 81.00 | 77.08 | .957 |
|  | *SD* | 22.17 | 23.35 |  | 25.03 | 27.13 |  | 17.51 | 22.63 |  |
|  | *Max* | 132 | 138.07 |  | 143 | 149.86 |  | 151 | 216.42 |  |
|  | *Min* | 14 | 12.34 |  | 8 | 2.20 |  | 37 | 16.22 |  |
| WPPSI-R/IV | *Mean* | 96.32 | 101.49 | .921 | 95.88 | 101.74 | .967 | 100.56 | 101.25 | .976 |
|  | *SD* | 27.15 | 25.89 |  | 27.43 | 31.50 |  | 21.32 | 26.31 |  |
|  | *Max* | 130 | 133.06 |  | 134 | 153.50 |  | 130 | 135.36 |  |
|  | *Min* | 31 | 35.08 |  | 40 | 39.53 |  | 44 | 30.63 |  |
| WISC-IV/V | *Mean* | 90.93 | 100.49 | .931 | 92.44 | 96.18 | .962 | 96.41 | 106.23 | .896 |
|  | *SD* | 22.22 | 23.97 |  | 22.46 | 23.07 |  | 21.41 | 30.98 |  |
|  | *Max* | 142 | 163.69 |  | 141 | 153.85 |  | 136 | 191.26 |  |
|  | *Min* | 40 | 58.36 |  | 45 | 51.83 |  | 45 | 58.36 |  |

Note. *r* = Pearson’s correlation coefficient

Table S3. Conditional multilevel growth models of ADOS total and domain scores for the DSM-IV cohort only

|  | Total | | Social Affect | | Repetitive & Restricted Behaviors | | |
| --- | --- | --- | --- | --- | --- | --- | --- |
| Time-Invariant Covariates | | | | | | | |
|  | Main Effect (SE) | Slope Interaction Effect (SE) | Main Effect (SE) | Slope Interaction Effect (SE) | Main Effect (SE) | | Slope Interaction Effect (SE) |
| Male | .14 (.57) | .32 (.11)^**^ | .14 (.59) | .29 (.10)^**^ | | .46 (.61) | .04 (.15) |
| Urban | 1.35 (.51)^**^ | -.24 (.10)^*^ | .53 (.52) | -.19 (.09)^*^ | 2.15 (.54)^***^ | | -.01 (.13) |
| SES | .58 (.31) | -.03 (.06) | .68 (.32)^*^ | -.05 (.06) | .36 (.34) | | .06 (.08) |
| Time-Varying Covariates^✝^ | | | | | | | |
|  | Main Effect (SE) | | Main Effect (SE) | | Main Effect (SE) | | |
| FSIQ | | | | | | | |
| *Person-level average (Level 2)* | -.05 (.01)^***^ | | -.04 (.01)^***^ | | -.04 (.01)^***^ | | |
| *Person-mean-centered (Level 1)* | -.06 (.03)^*^ | | -.07 (.03)^**^ | | .01 (.03) | | |
| *FSIQ*male* | .07 (.03)^**^ | | .07 (.02)^**^ | | .00 (.03) | | |
| *FSIQ*Urban* | -.04 (.02) | | -.03 (.02) | | -.04 (.03) | | |
| *FSIQ*SES* | -.01 (.02) | | -.02 (.01) | | .03 (.02) | | |
| VIQ | | | | | | | |
| *Person-level average (Level 2)* | -.05 (.01)^***^ | | -.04 (.02)^***^ | | -.04 (.01)^***^ | | |
| *Person-mean-centered (Level 1)* | -.03 (.02) | | -.04 (.02)^*^ | | -.00 (.02) | | |
| *VIQ*male* | .05 (.02)^**^ | | .05 (.02)^**^ | | .00 (.02) | | |
| *VIQ*Urban* | -.04 (.02)^*^ | | -.03 (.02)^*^ | | -.02 (.02) | | |
| *VIQ*SES* | -.00 (.01) | | -.01 (.01) | | .01 (.01) | | |
| NVIQ | | | | | | | |
| *Person-level average (Level 2)* | -.05 (.01)^***^ | | -.04 (.01)^***^ | | -.04 (.01)^***^ | | |
| *Person-mean-centered (Level 1)* | -.07 (.04)^*^ | | -.10 (.04)^**^ | | .04 (.05) | | |
| *NVIQ*male* | .08 (.04)^*^ | | .10 (.04)^**^ | | -.03 (.05) | | |
| *NVIQ*Urban* | -.03 (.03) | | -.01 (.03) | | -.04 (.03) | | |
| *NVIQ*SES* | -.03 (.02) | | -.04 (.02) | | .03 (.02) | | |

Note. ^*^*p* < .05, ^**^*p* < .01, ^***^*p* <.001; ^✝^FSIQ, VIQ, and NVIQ were included in separate models.

Figure S1. Model-estimated trajectories of ADOS total and domain scores


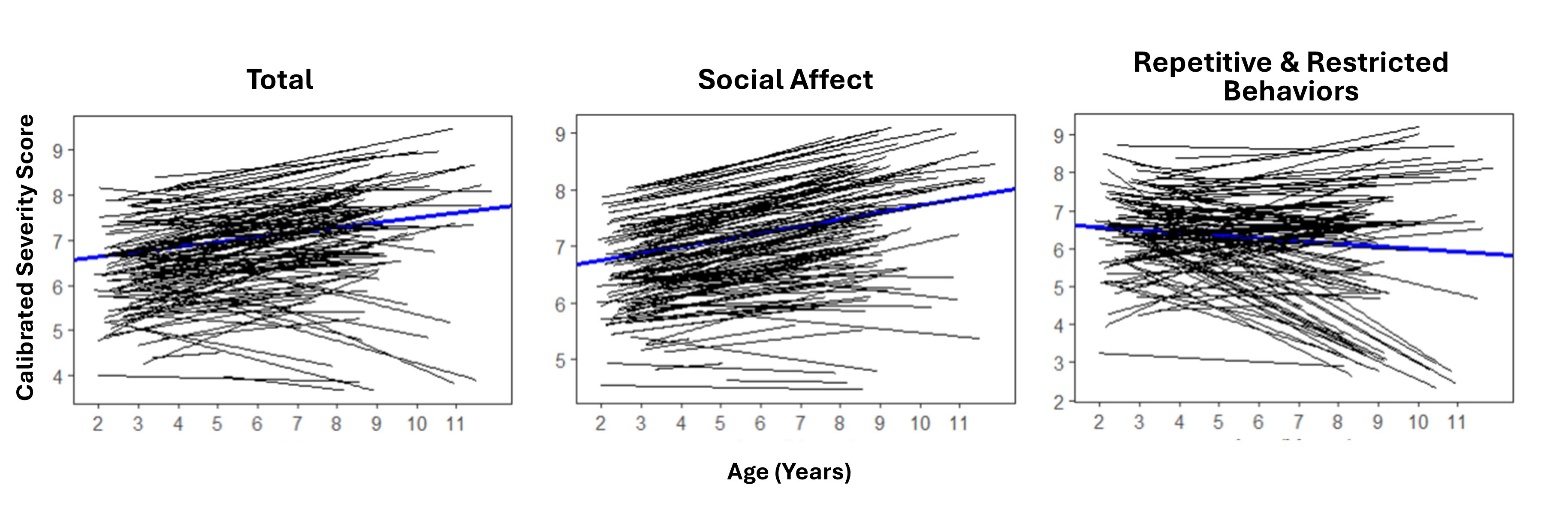


Note. Each black line represents an individual trajectory of calibrated severity scores over time. The thick blue line in each panel indicates the average trajectory across individuals.


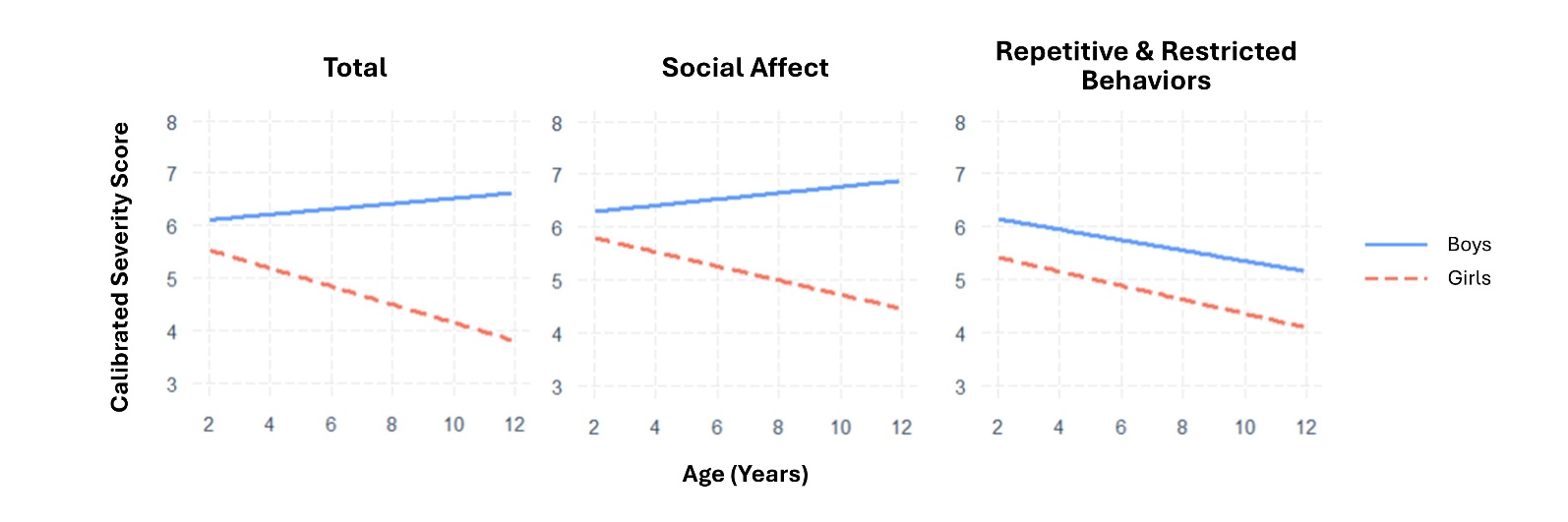
Figure S2. Model-estimated linear trajectories of ADOS total and domain scores by sex for the DSM-IV cohort only
